# Cytisine Exerts an Anti-Epileptic Effect via α7nAChRs in a Rat Model of Temporal Lobe Epilepsy

**DOI:** 10.3389/fphar.2021.706225

**Published:** 2021-06-24

**Authors:** Jing-jun Zheng, Teng-yue Zhang, Hong-tao Liu, Ze-xin Huang, Jing-mei Teng, Jing-xian Deng, Jia-gui Zhong, Xu Qian, Xin-wen Sheng, Ji-qiang Ding, Shu-qiao He, Xin Zhao, Wei-dong Ji, De-feng Qi, Wei Li, Mei Zhang

**Affiliations:** ^1^ Key Laboratory of Molecular Target and Clinical Pharmacology, Department of Clinical Pharmacology, School of Pharmaceutical Sciences and the Fifth Affiliated Hospital, Guangzhou Medical University, Guangzhou, China; ^2^ Department of Pharmacy, Shenzhen Kangning Hospital, Shenzhen Mental Health Center, Shenzhen, China; ^3^ Department of Neurosurgery, The First Affiliated Hospital of Jinan University, Guangzhou, China; ^4^ Department of Pharmacy, Maoming People’s Hospital, Maoming, China; ^5^ Center for Translational Medicine, The First Affiliated Hospital, Sun Yat-sen University, Guangzhou, China; ^6^ Department of Urology, Minimally Invasive Surgery Center, The First Affiliated Hop-ital of Guangzhou Medical University, Guangdong Key Laboratory of Urology, Guangzhou, China

**Keywords:** cytisine, temporal lobe epilepsy, neuroprotection, synaptic remodeling, cholinergic transmission, α7nAChRs

## Abstract

**Background and Purpose:** Temporal lobe epilepsy (TLE) is a common chronic neurological disease that is often invulnerable to anti-epileptic drugs. Increasing data have demonstrated that acetylcholine (ACh) and cholinergic neurotransmission are involved in the pathophysiology of epilepsy. Cytisine, a full agonist of α7 nicotinic acetylcholine receptors (α7nAChRs) and a partial agonist of α4β2nAChRs, has been widely applied for smoking cessation and has shown neuroprotection in neurological diseases. However, whether cytisine plays a role in treating TLE has not yet been determined.

**Experimental Approach:** In this study, cytisine was injected intraperitoneally into pilocarpine-induced epileptic rats for three weeks. Alpha-bungarotoxin (α-bgt), a specific α7nAChR antagonist, was used to evaluate the mechanism of action of cytisine. Rats were assayed for the occurrence of seizures and cognitive function by video surveillance and Morris water maze. Hippocampal injuries and synaptic structure were assessed by Nissl staining and Golgi staining. Furthermore, levels of glutamate, γ-aminobutyric acid (GABA), ACh, and α7nAChRs were measured.

**Results: **Cytisine significantly reduced seizures and hippocampal damage while improving cognition and inhibiting synaptic remodeling in TLE rats. Additionally, cytisine decreased glutamate levels without altering GABA levels, and increased ACh levels and α7nAChR expression in the hippocampi of TLE rats. α-bgt antagonized the above-mentioned effects of cytisine treatment.

**Conclusion and Implications:** Taken together, these findings indicate that cytisine exerted an anti-epileptic and neuroprotective effect in TLE rats via activation of α7nAChRs, which was associated with a decrease in glutamate levels, inhibition of synaptic remodeling, and improvement of cholinergic transmission in the hippocampus. Hence, our findings not only suggest that cytisine represents a promising anti-epileptic drug, but provides evidence of α7nAChRs as a novel therapeutic target for TLE.

## Introduction

Epilepsy is characterized by spontaneous seizures and is the second-most-prevalent neurological disease. Approximately 70 million people worldwide suffer from epilepsy (Singh and Trevick, 2016; Asadi-Pooya et al., 2017; Song et al., 2017). Anti-epileptic drugs (AEDs) remain as the primary treatment for epilepsy. Despite there being approximately 30 AEDs available with diverse molecular targets, one-third of patients still develop drug-resistant epilepsy, among which the most common type is temporal lobe epilepsy (TLE) (Asadi-Pooya et al., 2017; Wang and Chen, 2019). Most TLE patients not only exhibit the typical sign of spontaneous recurrent seizures (SRS), but also suffer from cognitive and memory impairments (Asadi-Pooya et al., 2017). In addition to psychological and physical stress, as well as exacerbation of mental disorders, the lives of epileptic patients are seriously affected by a much higher risk of disability and death (Robertson et al., 2015; Devinsky et al., 2018). Therefore, continued discovery of novel drug targets and promising drugs is imperative for improving TLE treatments.

The pathological mechanisms of epilepsy involve abnormal ion channels, inflammation, neuronal death, gliosis, and synaptic structural changes (Pitkänen and Lukasiuk, 2011; Pitkanen et al., 2015). It has long been considered that epilepsy is due to hyper-synchronous neuronal activity, during which the balance between excitation and inhibition is disrupted. Glutamate and γ-aminobutyric acid (GABA) are the most prevalent excitatory and inhibitory neurotransmitters, respectively, in the central nervous system (CNS), and play a pivotal role in epileptogenesis (Khazipov, 2016; Albrecht and Zielińska, 2017). Recently, increasing data have suggested that acetylcholine (ACh) and cholinergic neurotransmission participate in the pathophysiology of epilepsy (Ghasemi and Hadipour-Niktarash, 2015; Biagioni et al., 2019; Meller et al., 2019). Genetic studies have also demonstrated that mutations in neuronal nicotinic ACh receptors (nAChRs) are responsible for some specific forms of epileptic disorders, such as juvenile myoclonic epilepsy (JME) and autosomal-dominant nocturnal frontal lobe epilepsy (ADNFLE) (Rozycka et al., 2009; Rozycka et al., 2013; Weltzin et al., 2016; Villa et al., 2019). Neural nAChRs are extensively distributed in the CNS and represent a large family of ligand-gated ion channels constructed from combinations of α (α2–α10) and β (β2–β4) subunits (Gotti et al., 2006). Among them, homomeric α7 and heteromeric α4β2 pentameric nAChRs are predominant subtypes in the brain (Gotti et al., 2006). It has been reported that α7 and α4β2 subtypes of nAChRs might be linked to some idiopathic types of epilepsy, including JME and ADNFLE (Rozycka et al., 2009; Rozycka et al., 2013; Weltzin et al., 2016; Villa et al., 2019). Therefore, nAChR abnormalities may serve as a biomarker in epilepsy with a genetic background (Garibotto et al., 2019), suggesting that nAChRs may also be a potential target for the treatment of epilepsy.

Cytisine (C_11_H_14_N_2_O), a quinolizidine alkaloid extracted from the seeds of Cytisus laburnum, a natural plant of the Leguminosae family, is a full agonist of α7nAChRs and a partial agonist of α4β2nAChRs (Rouden et al., 2014). Historically, cytisine was initially used as an emetic, purgative, respiratory, analeptic, and diuretic agent in both Europe and North America (Paduszynska et al., 2018; Tutka et al., 2019). Presently, cytisine is widely applied for smoking cessation in Central and Eastern Europe (trade names: Tabex and Desmoxan) (Paduszynska et al., 2018; Tutka et al., 2019). There is a high systemic bioavailability of cytisine in healthy smokers taking this drug for smoking cessation, albeit with mild adverse reactions (Hajek et al., 2013). Accumulating evidence has shown that cytisine has neuroprotective effects in some neurological diseases such as Parkinson’s disease, depression, and cerebral injury induced by ischemia-reperfusion in animal models (Abin-Carriquiry et al., 2008; Han et al., 2016; Zhao et al., 2018). However, whether cytisine plays a role in the treatment of chronic spontaneous epilepsy remains unknown. Therefore, the present study investigated the effects and underlying mechanisms of cytisine in a rat model of TLE. Our results not only provide novel evidence of the use of cytisine as an efficacious drug for treating TLE, but further verify that nAChRs may serve as a therapeutic target for ameliorating epilepsy.

## Results

### Cytisine Reduces the Occurrence of SRS in Epileptic Rats

SRS emerged at an average of 12 days after SE induction in rats, which is consistent with our previous research (Qian et al., 2019). Cytisine treatment for three weeks after the onset of SRS significantly reduced the frequency of SRS without influencing seizure durations (Table 1). However, α-bgt inhibited this effect in cytisine-treated epileptic rats, which suggests that cytisine’s anti-epileptic effect involved α7nAChR signaling. Importantly, α-bgt administration alone did not affect SRS frequency in epileptic rats, nor did it induce any adverse reactions in normal rats.

**TABLE 1:** Cytisine reduces the frequency of SRS in epileptic rats.

Groups	Seizurs/week (times)	Seizurs score	Seizure durations(s)
Normal	0	—	0
Epilepsy	5.17 ± 0.48	IV/V	23.56 ± 4.22
Ep + Cyt	2.33 ± 0.56^#^	IV/V	21.38 ± 3.91
Ep + Cyt + α-bgt	4.83 ± 0.91 ^$^	IV/V	20.31 ± 3.72
Ep + α-bgt	5.83 ± 0.40	IV/V	26.73 ± 5.65
Norm + α-bgt	0	—	0

Experimental rats received cytisine (2 mg/kg/d) and α-bgt (1 μg/kg/d) by i.p injection for three weeks. The numbers and durations of SRS were recorded. Cyt is the abbreviation of cytisine, Ep is the abbreviation of epilepsy, and Norm is the abbreviation of normal. Normal and Norm + α-bgt rats did not have any seizures. The statistical results of the remaining four groups are as follows (seizures/week): F (3,36) = 15.422, n = 10, epilepsy vs. epilepsy + Cyt, ^#^p < 0.05, epilepsy + Cyt vs. epilepsy + Cyt + α-bgt, ^$^p < 0.05.

### Cytisine ameliorates Cognitive Dysfunction in Epileptic Rats

**FIGURE 1:**
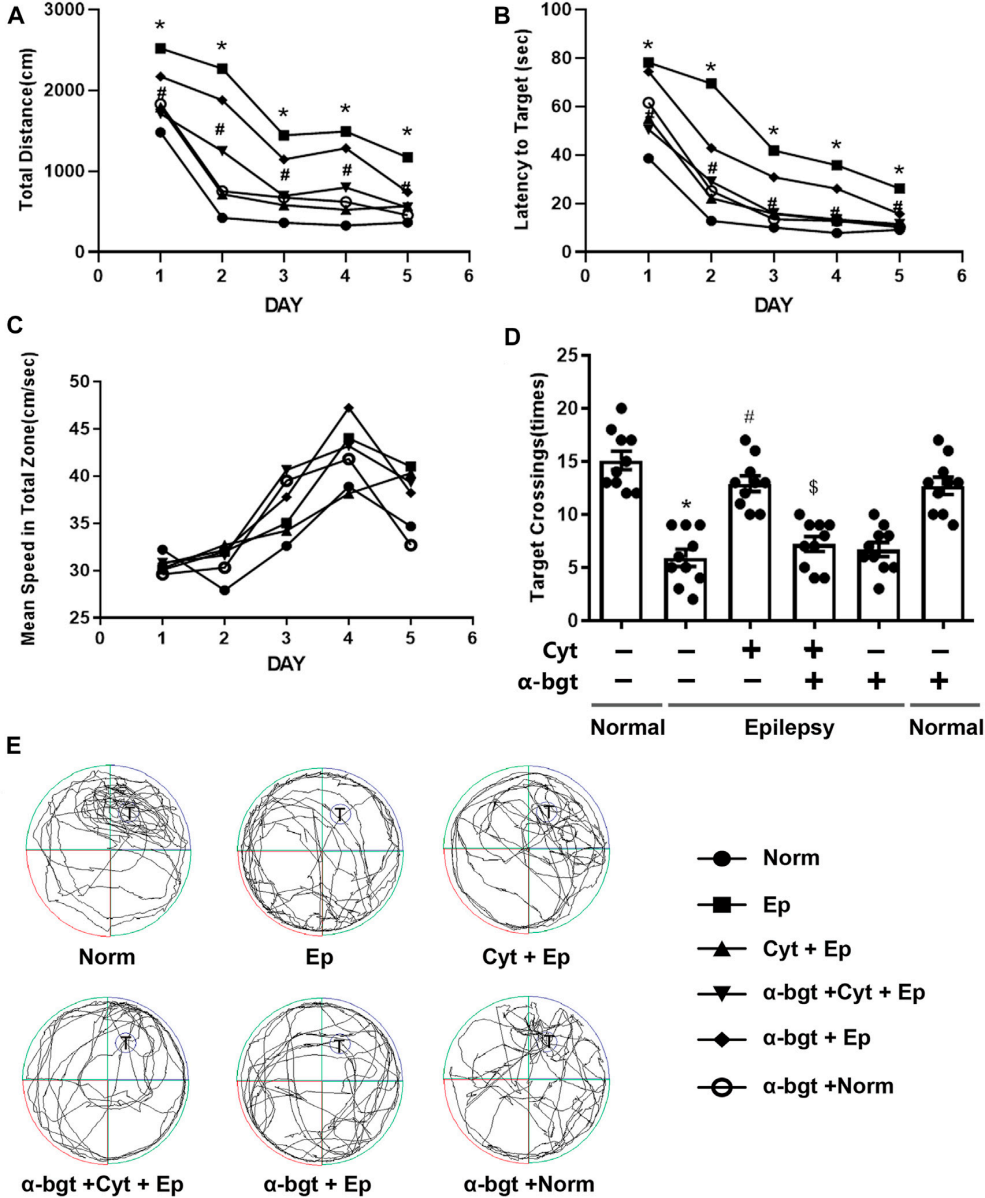
*Cytisine ameliorates deficits in learning and memory in TLE rats*. The effects of cytisine on learning and memory in rats were evaluated using the Morris water maze. **A**–**C:** Acquisition training. **A:** Swim path distance. **B:** Latency to reach underwater platform. **C:** Mean swim speed. **D**–**E:** Probe trial. **D:** The number of crossing over the target quadrant. **E:** Searching trajectories for the target quadrant (T: target). Statistical results are as follows: F (5,54) = 26.017, *n* = 10; normal vs. epilepsy, *p < 0.05; epilepsy vs. epilepsy + Cyt, ^#^ *p* < 0.05; epilepsy + Cyt vs. epilepsy + Cyt + α-bgt, ^**$**^ *p* < 0.05.

Learning and memory were assessed using the Morris water maze. The path length, latency to reach the target platform, and average swimming speed were recorded during all 5 days of training (Figures 1A–C). Epileptic rats showed decreased learning in searching for the underwater platform as compared to that of the normal group, which was manifested as an increased travel distance and latency to reach the target platform during the training trials (Figures 1A,B). There was no significant difference in swimming speed among the groups during the 5 days of training (Figure 1C). Epileptic rats spent more time searching for the target quadrant and had a reduced number of crossing over the platform area during the probe trial compared with these parameters in normal rats (Figures 1D,E). Cytisine treatment significantly shortened both the distance and latency to reach the target platform (Figures 1A,B), and increased the number of crossing over the target platform in epileptic rats compared to those treated with vehicle (Figures 1D,E). These results indicated that impaired learning abilities in epileptic rats were rescued by cytisine treatment. Furthermore, α-bgt antagonized cytisine’s effect on cognitive deficits in TLE rats. Importantly, α-bgt used alone did not influence learning and memory in either epileptic rats or normal rats.

### Cytisine attenuates Hippocampal Injuries in Epileptic Rats

**FIGURE 2:**
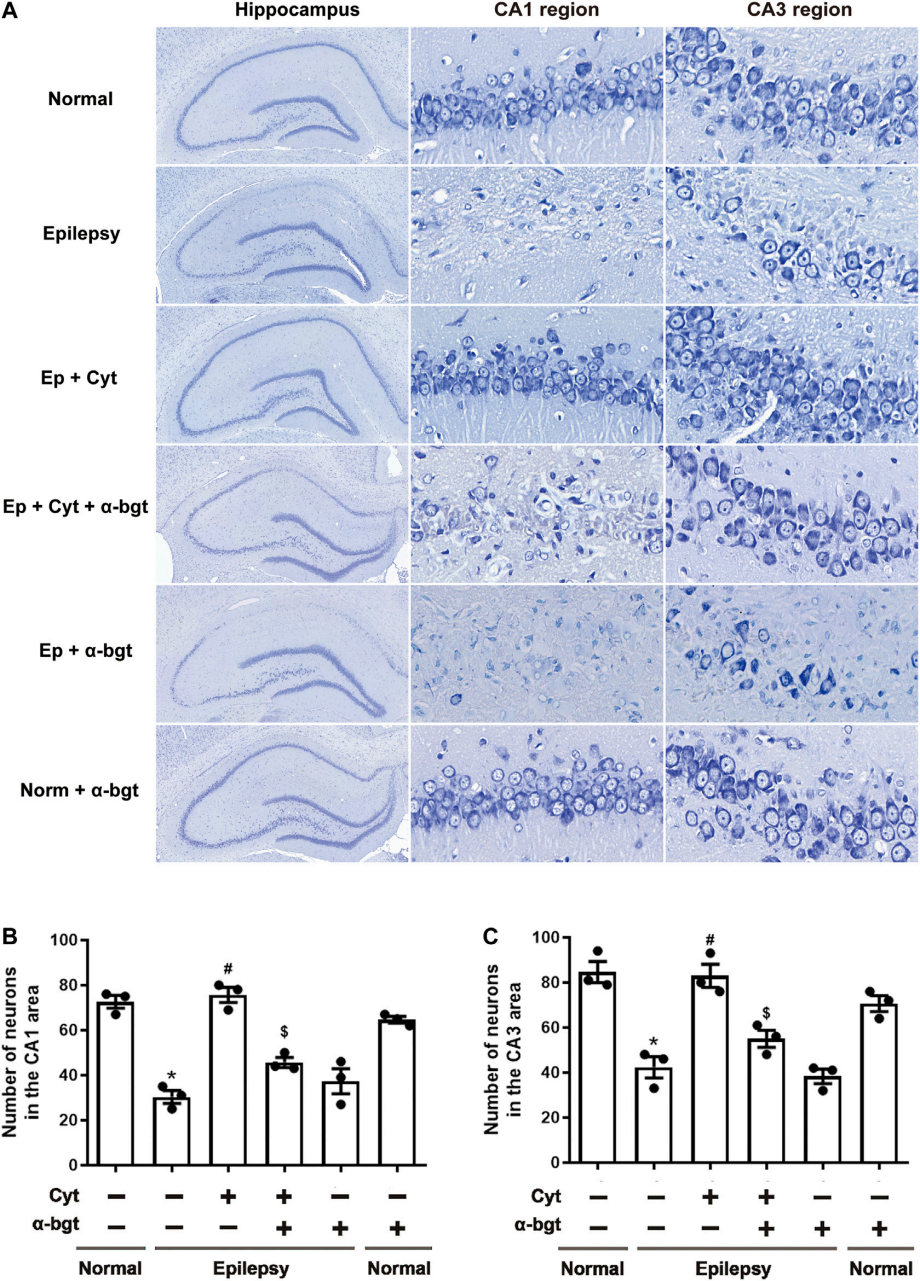
*Cytisine attenuates hippocampal damage in epileptic rats.* Nissl staining was used to assess neuronal damage in hippocampal regions. **A:** Nissl staining of the entire hippocampus (5×). CA1 and CA3 regions of the hippocampus (90×). **B:** Quantitative analysis of pyramidal neurons in CA1 regions. Statistical results are as follows: **F** (5,12) = 33.59, *p* < 0.05, *n* = 3; normal vs. epilepsy, *p < 0.05; epilepsy vs. epilepsy + Cyt, ^**#**^ *p* < 0.05; epilepsy + Cyt vs. epilepsy + Cyt + α-bgt, ^**$**^ *p* < 0.05. **C:** Quantitative analysis of pyramidal neurons in CA3 regions. Statistical results are as follows: **F** (5,12) = 22.618, *p* < 0.05, *n* = 3; normal vs. epilepsy, *p < 0.05; epilepsy vs. epilepsy + Cyt, ^**#**^ *p* < 0.05; epilepsy + Cyt vs. epilepsy + Cyt + α-bgt, ^**$**^ *p* < 0.05.

Neuronal damage in the hippocampus was examined via Nissl staining. Our results showed that pyramidal neurons were aligned regularly and exhibited intact structures with clear nucleoli in hippocampal CA1 and CA3 regions of normal rats (Figure 2A). CA1 regions exhibited destruction of the layered structure of pyramidal neurons with an evident neuronal loss, and CA3 regions exhibited a disordered arrangement of pyramidal neurons with an atactic shape, opaque cytoplasm, and shrinking nuclei in the hippocampi of epileptic rats (Figure 2A). Cytisine treatment significantly attenuated the epileptic-induced loss of pyramidal neurons and mitigated these pathological changes in both CA1 and CA3 regions of the hippocampus (Figures 2B,C). Furthermore, α-bgt partially abolished cytisine’s neuroprotective effect on hippocampal neurons. Importantly, α-bgt used alone did not influence hippocampal structures in either normal or epileptic rats.

### Influences of Cytisine on Neurotransmitters in the Hippocampus of Epileptic Rats

**FIGURE 3:**
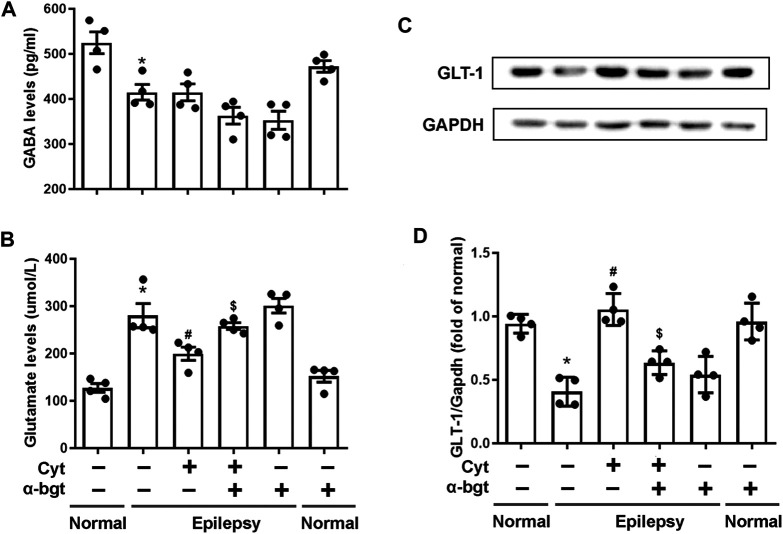
*Influences of cytisine treatment on neurotransmitter levels in the hippocampi of epileptic rats.* **A**–**B:** GABA and glutamate levels in the hippocampus were measured by colorimetry. Statistical results are as follows: GABA: **F** (5,18) = 11.924, *p* < 0.05, *n* = 4; normal vs. epilepsy, *p < 0.05; epilepsy vs. epilepsy + Cyt, *p* > 0.05; epilepsy + Cyt vs. epilepsy + Cyt + α-bgt, *p* > 0.05; Glu: **F** (5,18) = 21.889, *p* < 0.05, *n* = 4; normal vs. epilepsy, *p < 0.05; epilepsy vs. epilepsy + Cyt, ^#^ *p* < 0.05; epilepsy + Cyt vs. epilepsy + Cyt + α-bgt, ^**$**^ *p* < 0.05. **C**–**D:** GLT-1 expression in the hippocampus was analyzed by Western blotting.Statistical results are as follows: GLT-1:F (5,18) = 19.741,*p* < 0.05, *n* = 4; normal vs. epilepsy, *p < 0.05; epilepsy vs. epilepsy + Cyt, ^#^ *p* < 0.05; epilepsy + Cyt vs. epilepsy + Cyt + α-bgt, ^**$**^ *p* < 0.05.

Glutamate and GABA are essential for maintaining a balance of neuronal excitation and inhibition within the brain. Our results demonstrated that glutamate levels were increased and GABA levels were decreased in the hippocampi of epileptic rats. Cytisine treatment remarkably decreased glutamate levels without altering GABA levels obviously (Figures 3A,B). GLT-1 is a glutamate transporter that is responsible for reuptake of excess glutamate from the synaptic cleft. To further determine changes related to epileptic-induced alterations in glutamate levels, the expression of GLT-1 in the hippocampus was assessed. We found that the expression of hippocampal GLT-1 was decreased in epileptic rats and that this decrease was reversed by cytisine compared to that of vehicle treatment, the effect of which may have accelerated reuptake of glutamate (Figures 3C,D). Furthermore, α-bgt inhibited cytisine’s effects on glutamate level and GLT-1 expression, but GABA levels were not changed. However, α-bgt used alone did not influence neurotransmitters levels or GLT-1 expression in either normal or epileptic rats.

### Cytisine inhibits Synapse Remodeling in the Hippocampi of Epileptic Rats

**FIGURE 4:**
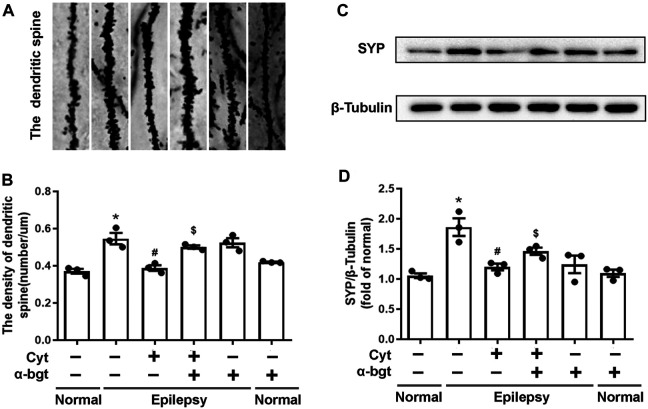
*Cytisine inhibits synaptic remodeling in the hippocampi of epileptic rats.* **A:** Dendritic spines dyed by Golgi staining in the dentate gyrus of the hippocampus. **B:** Quantitative analysis of the densities of dendritic spines. Statistical results are as follows: **F** (5,12) = 16.685, *p* < 0.05, *n* = 3; normal vs. epilepsy, *p < 0.05; epilepsy vs. epilepsy + Cyt, ^#^ *p* < 0.05; epilepsy + Cyt vs. epilepsy + Cyt + α-bgt, ^**$**^ *p* < 0.05. **C**–**D:** The expression of synaptophysin in the hippocampus was analyzed by Western blotting. Statistical results are as follows: **F** (5,12) = 10.802, *p* < 0.05, *n* = 3; normal vs. epilepsy, *p < 0.05; epilepsy vs. epilepsy + Cyt, ^#^ *p* < 0.05; epilepsy + Cyt vs. epilepsy + Cyt + α-bgt, ^**$**^ *p* < 0.05.

Synapse remodeling occurs as a result of epileptogenesis. Hence, synaptic structures were assessed to investigate whether cytisine’s anti-epileptic effect involved synaptic remodeling. The densities of dendritic spines in epileptic rats were increased significantly compared to those in normal rats, suggesting synaptic remodeling in the hippocampus of epileptic rats. Cytisine treatment reversed this alteration by reducing the number of dendritic spines (Figures 4A,B). Synaptophysin, a synapse-associated protein, participates in synaptic remodeling. Our results showed that synaptophysin expression was increased in the hippocampi of epileptic rats, and we found that cytisine treatment suppressed this change (Figures 4C,D). Furthermore, α-bgt antagonized cytistine-induced inhibition in the numbers of dendritic spines, and simultaneously abolished cytisine-induced suppression of synaptophysin expression. However, α-bgt used alone did not have any impact on the density of dendritic spines or synaptophysin expression in either normal or epileptic rats.

### Cytisine Strengthens Cholinergic Transmission in the Hippocampi of Epileptic Rats

**FIGURE 5:**
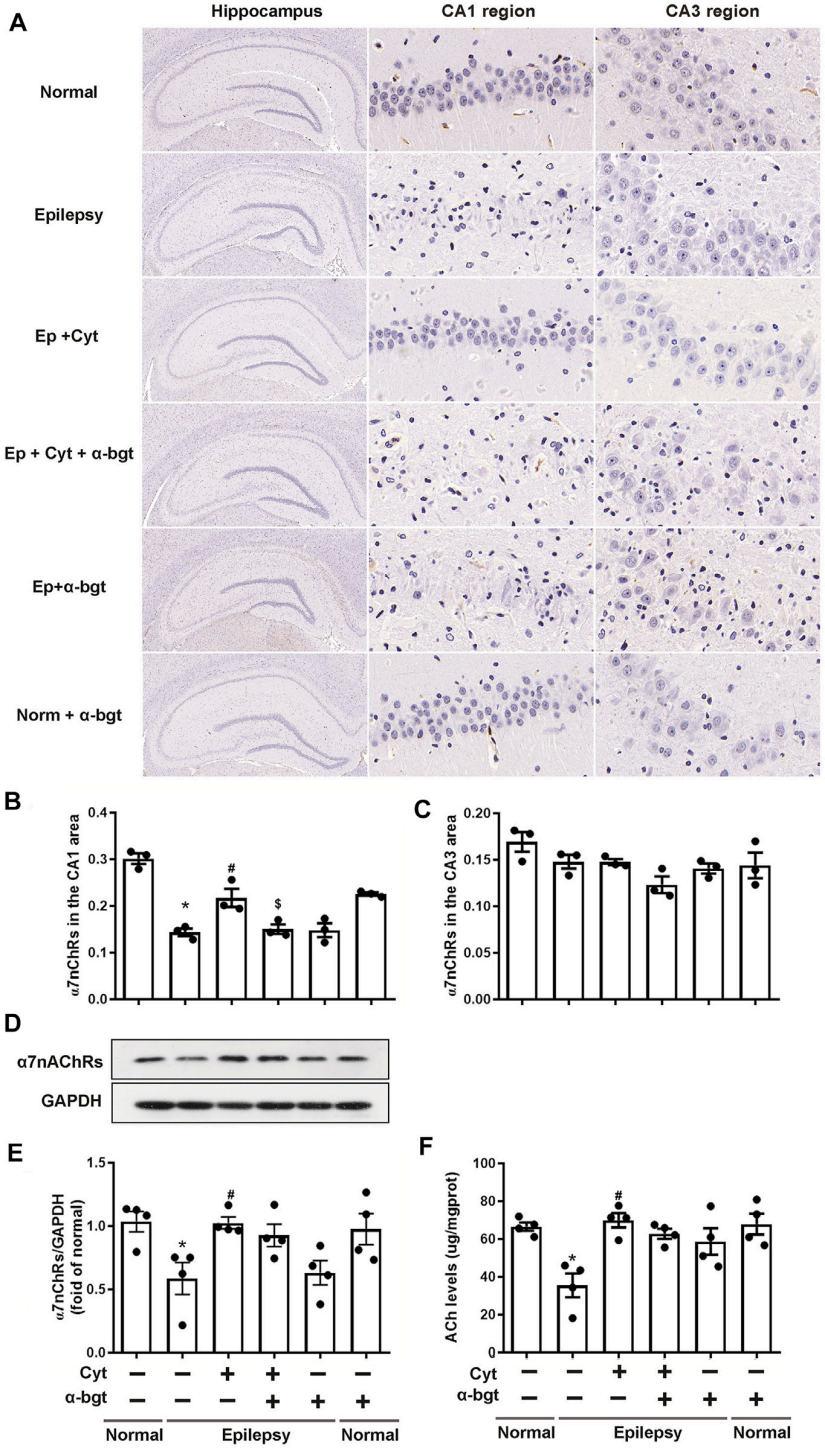
*Cytisine enhances cholinergic transmission in the hippocampi of epileptic rats.* **A**–**C:** The expression of α7nAChRs in the hippocampus was stained by immunohistochemistry. Statistical results are as follows: **F** (5,12) = 26.033, *p* < 0.05, *n* = 3; normal vs. epilepsy, *p < 0.05; epilepsy vs. epilepsy + Cyt, ^#^ *p* < 0.05; epilepsy + Cyt vs. epilepsy + Cyt + α-bgt, ^**$**^ *p* < 0.05. **D**–**E:** The expression of α7nAChRs in the hippocampus was analyzed by Western blotting. Statistical results are as follows: F (5,18) = 4.271, *p* < 0.05, *n* = 4; normal vs. epilepsy, *p < 0.05; epilepsy vs. epilepsy + Cyt, ^#^ *p* < 0.05. **F:** ACh levels in the hippocampus were measured by colorimetry. Statistical results are as follows: **F** (5,18) = 6.705, *p* < 0.05, *n* = 4; normal vs. epilepsy, *p < 0.05; epilepsy vs. epilepsy + Cyt, ^#^ *p* < 0.05.

Cholinergic transmission is altered as a result of epileptogenesis. Thus, ACh levels and α7nAChR expression were measured in order to investigate whether cytisine’s therapeutic amelioration of epilepsy was associated with cholinergic transmission. Our experiments showed that ACh levels were significantly decreased in the hippocampi of epileptic rats (Figure 5F). Accordingly, both hippocampal slices and tissue homogenates from epileptic rats showed decreased expression of α7nAChRs (Figures 5A–E). Moreover, cytisine treatment increased ACh levels and α7nAChR expression. α-bgt partly blocked cytisine’s effects on ACh levels or α7nAChR expression, but showing no significance. However, α-bgt used alone increased ACh levels without affecting α7nAChR expression in epileptic rats, and did not influence either of these parameters in normal rats.

## Discussion

Our present study demonstrated that cytisine treatment for three weeks after the onset of spontaneous seizures in TLE rats significantly reduced the frequency of SRS, mitigated deficits in learning and memory, ameliorated hippocampal damage, and inhibited synaptic remodeling. These findings indicate that cytisine exerted anti-epileptic and neuroprotective effects in TLE rats. Moreover, these findings represent the first demonstration that cytisine mitigates TLE in rats.

Hyperactivity of glutamatergic function and insufficient GABAergic neurotransmission has been regarded as the most important mechanisms in epileptic seizures (Khazipov, 2016; Albrecht and Zielińska, 2017). Based on this canonical perspective, current AEDs play a therapeutic role via either inhibiting excitatory drive or strengthening inhibitory drive. Some conventional antiepileptic drugs such as sodium valproate, carbamazepine, and oxcarbazepine work in this manner. The present study showed that the occurrences of spontaneous seizures in TLE rats were decreased following cytisine treatment. Further investigation indicated that cytisine significantly reduced glutamate levels in hippocampus of TLE rats, which may have accounted for the concomitant reduction in SRS following cytisine treatment. However, it has been shown that activation of α7nAChRs by nicotine enhanced glutamate release in hippocampal pyramidal neurons (Cheng and Yakel, 2015). As an agonist of α7nAChRs, present study demonstrated that cytisine reduced glutamate levels in hippocampus of TLE rats. For this inconsistency, we speculated that it may be due to the effect of cytisine on neuroglial cells. Besides neuronal expression, α7nAChRs are also expressed on non-neuronal cells such as astrocytes in the hippocampal CA1 region (Shen and Yakel, 2012)**.** The clearance of glutamate from synapse is performed by neuroglia via glutamate transporters, which is mediated by activation of α7nAChRs (Morioka et al., 2014). Compared to neuroglial cells, hippocampal pyramidal neurons are more vulnerable to insults under pathological conditions, which also has been shown in our present study. Epileptic seizures initially result in damages to hippocampal neurons, accompanied by a decrease of α7nAChRs that weakened its role in modulating neurotransmitters release. Under this circumstance, neuroglial cells may play a more important role in glutamate metabolism. Glutamate reuptake at the synaptic cleft is mainly mediated by GLT-1 (Rimmele and Rosenberg, 2016). Our present study showed that cytisine increased the expression of GLT-1 in the hippocampus of TLE rats. Accordingly, this may result in accelerated glutamate clearance and decreased synaptic glutamate levels. Although cytisine activates both α7 and α4β2 subtypes of nAChRs, it is a full agonist of α7nAChRs, suggesting that high-efficacy activation of α7nAChRs may be more important for cytisine’s therapeutic effects (Rouden et al., 2014; Tutka et al., 2019). Hence, in our present study, the α7nAChR-specific blocker, α-bgt, was used to determine its influence on cytisine’s effects. In our experiments, methyllcaconitine (MLA), a reversible antagonist of α7nAChR, has not been selected as the α7nAChR blocker for its affinity, albeit low, to α3β4 and α4β2 subtypes (Drasdo et al., 1992). We found that α-bgt antagonized not only anti-epileptic effects, but also effects on glutamate reuptake, induced by cytisine treatment, indicating that α7nAChR signaling is involved in cytisine’s anti-epileptic effects. It should be mentioned that α-bgt administration alone did not affect SRS frequency in epileptic rats, nor did it induce any adverse reactions in normal rats. In our present study, a dose of 1 μg/kg α-bgt was selected for treatment, and this dose was also used for other previous studies (Gao et al., 2010; Du et al., 2013). A higher dose of α-bgt (3–5 μg/kg) was often used to induce animal model for myasthenia gravis in which the main symptoms manifested as facial muscle weakness, showing no obvious toxic effects (Plomp et al., 1994). It should be noted that cytisine did not alter GABA levels in the hippocampus of TLE rats at present study. Although α7nAChRs are also involved in the functional modulation of GABAergic neurons, cholinergic neuromodulation in GABAergic interneurons is extremely complicated, involving in multiple factors such as network oscillation, et al. (Lawrence, 2008). In addition, damages of hippocampal GABAergic neurons during epileptogenesis may partly abolished the modulation of α7nAChRs activation on neurotransmitter release. Therefore, the exact effects of cytisine on GABAergic transmission require further investigation.

The hippocampus is an essential region in the brain that is closely associated with cognition and memory. Hippocampal neuronal injury is the primary neuropathological feature of TLE that contributes to cognitive and memory impairments (Titiz et al., 2014). Our previous study showed that lithium- and pilocarpine-induced epileptic rats exhibited typical traits of TLE accompanied by cognitive deficits (Qian et al., 2019). Our present study indicated that cytisine treatment reduced epileptic-mediated loss of pyramidal neurons in the CA1 and CA3 regions of the hippocampus and mitigated deficits in learning and memory in TLE rats. Several other studies have demonstrated that cytisine confers neuroprotection against cytotoxicity induced by beta amyloid and N-methyl-D-aspartate in both cortical neurons *in vitro* and in a mouse model of cerebral ischemia-reperfusion injury (Kihara et al., 1998; Li et al., 2013; Zhao et al., 2018). Similarly, cytisine has been shown to exhibit neuroprotection of dopaminergic neurons in a mouse model of Parkinson’s disease (Ferger et al., 1998; Abin-Carriquiry et al., 2008). Consistent with these previous findings, our present study revealed that cytisine also exerted neuroprotection in TLE rats. In inconsistent to our present results, in previous studies cytisine attenuated neuroprotection and anticonvulsive activity when combined with antiepileptic drugs in electroshock and 6-Hz stimulation-induced mouse model (Tutka et al., 2013; Tutka et al., 2017). This discrepancy may be due to differences in the types of epileptic models and the manner of cytisine treatments. In our present study, we found that α-bgt antagonized cytisine’s neuroprotective effects in TLE rats, suggesting a role for α7nAChR signaling.

Synaptic remodeling occurs during the development of the brain and shares common features with epileptogenesis (Bozzi et al., 2012). Neuronal loss and abnormal synaptic contacts induced by seizures result in aberrant neuronal network in both animal models of epilepsy and in human TLE, which may be associated with enhancement of synaptic efficiency and may play an important role in epileptogenesis (Leite et al., 2005; Lillis et al., 2015). Dendritic spines, the dendritic arborizations of neurons, are key synaptic structures and postsynaptic targets of excitatory synapses in the CNS. Abnormally-high numbers of dendritic spines strengthen synaptic transmission and are attributed to neuronal hyperexcitability in epilepsy (Ma et al., 2013; Janz et al., 2018). Our present study demonstrated that numbers and densities of dendritic spines were significantly increased in the hippocampi of TLE rats, and cytisine treatment reduced this epileptic-induced augmentation of dendritic spines. Therefore, cytisine treatment may have inhibited epileptic-mediated formation of abnormal excitability in the hippocampus, which may have resulted in reducing SRS in TLE rats. Synaptophysin is a vesicular protein that is widely expressed in axonal terminals and is closely related to synaptic structure and function (Thiel, 1993). As an important marker for synaptic activity, synaptophysin participates in synaptogenesis and synaptic remodeling (Masliah et al., 1991; Thiel, 1993). In our present study, we found that cytisine reduced epileptic-induced upregulation of synaptophysin in the hippocampi of TLE rats, suggesting that inhibition of synaptic reorganization may represent one of the mechanisms underlying cytisine’s anti-epileptic effects. In line with the above results, we found that α-bgt antagonized cytisine’s reduction of synaptic remodeling in TLE rats.

Accumulating evidence suggests that abnormal central cholinergic signaling is involved in the pathophysiology of epilepsy (Hamilton et al., 1997; Ferencz et al., 2001; Ghasemi and Hadipour-Niktarash, 2015; Biagioni et al., 2019; Meller et al., 2019). Cholinergic neurons serve as an important part of the CNS in terms of regulating neuronal excitability, modulating synaptic transmission, and inducing synaptic plasticity (Picciotto et al., 2012). Recently, a number of studies have demonstrated that ACh and cholinergic neurotransmission are involved in epileptogenesis (Ghasemi and Hadipour-Niktarash, 2015; Biagioni et al., 2019; Meller et al., 2019). Genetic studies have shown that mutations in neuronal nAChRs are responsible for some forms of idiopathic epilepsy (Rozycka et al., 2013; Weltzin et al., 2016; Garibotto et al., 2019; Villa et al., 2019). In our present study, we found that both ACh levels and α7nAChR expression were decreased in the hippocampi of TLE rats, while treatment with cytisine reversed these alterations, indicating that cytisine ameliorated epileptic-induced deficits in cholinergic transmission in TLE rats. A number of studies have shown that cholinergic transmission plays a role in epilepsy. The cholinergic system exerts powerful antiepileptic effects in hippocampal-kindling epileptogenesis (Ferencz et al., 2001). Additionally, degeneration of cholinergic nuclei in the basal forebrain is associated with focally-induced seizures (Biagioni et al., 2019). A few conventional antiepileptic drugs, such as carbamazepine and zonisamide, increase basal ACh levels via facilitating release in various brain regions (Mizuno et al., 2000; Zhu et al., 2002). The recent study has shown that ACh levels are decreased in the hippocampi of two rat models of TLE during chronic epileptic states (Meller et al., 2019). Similarly, our present study not only further confirmed that cholinergic transmission was involved in epilepsy, but also suggests that cytisine exerted antiepileptic effects by compensating for cholinergic deficiency in epileptic rats. Previous studies have shown that nAChR agonists induce up-regulation of nAChR and facilitate the release of ACh (Beani et al., 1985; Nordberg et al., 1989; Madhok et al., 1995; Doura et al., 2008), which are coincide with our data that the effects of cytisine on nAChR expression and ACh level in epileptic rats in present study. Despite its role as the primary excitatory neurotransmitter in periphery, ACh mainly plays a neuromodulatory role in the brain (Picciotto et al., 2012). As a result, it has been determined that ACh does not exert merely an excitatory or inhibitory action in epilepsy. Therefore, we speculated that decreased ACh and α7nAChRs in the brains of epileptic rats may weaken its neuroregulation, leading to an imbalance of excitation and inhibition, while cytisine produce an antiepileptic effect by restoring abnormal cholinergic regulation. However, in our present study, we found that α-bgt did not significantly block cytisine’s effects on ACh levels and α-bgt used alone increased ACh levels in brain of epileptic rats. Some previous studies have shown that chronic α-bgt treatment facilitates ACh release in motor nerve endings (Miledi et al., 1978; Plomp et al., 1994; Plomp and Molenaar, 1996). Consequently, α-bgt-induced the elevation of ACh levels partly counteracted its effect on cytisine’s action as an antagonist of α7nAChRs in brains of TLE rats.

In light of its involvement in epilepsy, neuronal nAChRs represent a novel target for therapy of epilepsy (Löscher et al., 2003; Ghasemi and Hadipour-Niktarash, 2015). A pre-clinical study has demonstrated that nAChR antagonists are beneficial in different epileptic animal models (Ghasemi and Hadipour-Niktarash, 2015). Conventional antiepileptic drugs, such as carbamazepine, zonisamide, and lamotrigine, exert anticonvulsive effects via blockade of α4β2nAChRs in some genetic models of epilepsy (Picard et al., 1999; Provini et al., 1999; Zheng et al., 2010). Our present study showed that cytisine exerted an antiepileptic effect by activating α7nAChRs in TLE rats. This result is contrary to the conventional understanding of how nAChRs antagonists work as antiepileptic agents. The use of nAChR antagonists for antiepileptic drug development has been controversial because such agents exhibit disparate antiepileptic effects across different animal models (Löscher et al., 2003). For instance, neuronal nAChR antagonists exert potent anticonvulsive effects on generalized epileptic seizures but show less robust effects on complex partial seizures (Löscher et al., 2003). Our present study was conducted in TLE rats, which is a complex partial-seizure model, and we investigated the role of α7nAChRs in the treatment of epilepsy. Thus, differences in epilepsy models and nAChR subtypes may partly account for the inconsistent results between our present study and some previous studies. Furthermore, in line with our present findings a recent study has demonstrated that the α7nAChR agonist, choline chloride, ameliorates seizure severity, memory impairment, and depression in a pentylenetetrazole-kindled mouse model of epilepsy (Sharma et al., 2020).

α7nAChRs are non-selective cation channels, but have a high Ca^2+^ permeability (Fucile et al., 2003). Intracellular Ca^2+^ signals contribute to the regulation of neurotransmitter release and neuronal plasticity (Role and Berg, 1996; Broide and Leslie, 1999). As a result, α7nAChRs may serve a distinct role in modulating these events. Our present results demonstrated that α7nAChR activation may be an important mechanism of cytisine’s anti-epileptic actions by affecting neurotransmitter release and synaptic remodeling. In its use as a medicine for smoking cessation, cytisine possesses good pharmacokinetics and mild adverse reactions such as nausea, vomiting, and dry mouth (Jeong et al., 2015; Paduszynska et al., 2018; Tutka et al., 2019). Moreover, cytisine has advantages in terms of its low cost and high tolerability, making it a promising agent in clinical applications (Rouden et al., 2014; Paduszynska et al., 2018). However, our present study only focused on the involvement of α7nAChRs in cytisine’s antiepileptic effects, as we did not investigate any potential role of the α4β2 subtype in our study. Hence, further studies are required to determine whether α4β2 nAChRs also play a role in cytisine’s antiepileptic effects. Additionally, the specific molecular mechanisms by which cytisine interacts with α7nAChRs requires elucidation in future studies *in vitro*.

## Materials and Methods

### Experimental Animals

#### Animals

Male Sprague-Dawley (200 ± 20 g) rats were purchased from the Experimental Animal Centre of Guangzhou University of Chinese Medicine (SCXK YUE 2013–0,034). Rats were bred under standard laboratory conditions under a 12 h light/dark cycle at 22 ± 2°C, with food and water provided ad libitum. Rats were fed habitually for one week before experiments. All procedures were conducted according to the National Institutes of Health Guidelines for Animal Research and were approved by the Ethics Committee for Animal Research at Guangzhou Medical University.

### Rat Model of Temporal Lobe Epilepsy

We established a pilocarpine-induced epileptic rat model in our present study according to a previous protocol (Qian et al., 2019). A total of 60 male rats were first treated with lithium chloride (127 mg/kg, Sigma-Aldrich, St. Louis, MO, United States ) and were then subsequently administered pilocarpine hydrochloride 18–20 h later (30 mg/kg, Sigma-Aldrich) via intraperitoneal injection. Atropine sulfate (1 mg/kg, King York, China) was used to reduce peripheral cholinergic side effects at 30 min before pilocarpine injection. Additional pilocarpine hydrochloride (10 mg/kg) was administered every 30 min until the development of status epilepticus (SE) for rats showing no occurrence following the first injection. The evoked seizures were scored according to the Racine scale. Seizures were classified as following: stage I, mouth and facial automatism; stage II, nodding or wet dog shaking; stage III, forelimb clonus; stage IV, standing and bilateral forelimb clonus; stage V, generalized clonic seizures and falls. Only rats with stage IV or stage V seizures were included in our experiment. 54 rats developed to status epilepticus. Control rats received the same treatment with vehicle. Rats sustained with SE for 60 min were then administered diazepam (10 mg/kg, King York, China) to terminate seizures. Intensive care including warming and oral injections of sugar saline to reduce the mortality rates of rats. Among 54 rats with epileptic seizures, 13 rats died in the acute phase. Survived animals were used in the following experiment and survival rate of rats were about 76%. The occurrence of SRS began approximately two weeks after SE.

### Animal Groupings and Drug Treatments

A total of 20 normal rats were randomly divided into two groups (10 rats per group): normal group and normal + α-bgt group. A total of 40 epileptic rats were randomly divided into four groups (10 rats per group): epilepsy group; epilepsy + cytisine group; epilepsy + cytisine + α-bgt group; epilepsy + α-bgt group. The α7nAChR-specific blocker, α-bgt, was employed to investigate the mechanism of action of cytisine. Because of its biotoxicity, α-bgt was administered alone to observe any possible adverse reactions in control and epileptic rats. Cytisine (Macklin, China) and α-bgt (Cayman, United States ) were dissolved in saline. Cytisine was administered daily via intraperitoneal injection at 2 mg/kg/d for three weeks after the onset of SRS, and α-bgt was injected intraperitoneally at a dose of 1 μg/kg/d at 30 min before cytisine injection.

Other rats received saline as a control. On accounting of its wide using, cytisine has not been administered alone to control rats in present experiment. All 10 rats received video monitoring and Morris water maze test. For Nissl staining and immunohistochemistry analysis, three rats were included, and three rats were used for Golgi staining. The remaining four rats were applied for measurement of neurotransmitters and western blotting assay.

### Monitoring of Spontaneous Recurrent Seizures

Rats were continuously video-monitored in transparent cages via a four-camera system (Hikvision, China). The occurrence, severity, and duration of spontaneous seizures were evaluated blindly by an observer, and stage IV seizures or above (as determined by the Racine scale) were recorded. Seizure-like activities in rats were confirmed by another observer in order to avoid bias.

### Morris Water Maze

The Morris water maze was carried out to evaluate learning and memory in rats. This test assesses place-mediated navigation and spatial exploration. The system consisted of a circular pool (150 cm diameter and 50 cm depth) and pool was filled with 30 cm depth of water (22–24°C) made opaque by the addition of non-toxic white paint. The pool was divided into four quadrants and a hidden platform with a diameter of 12 cm was located in a fixed target quadrant 2 cm beneath the water surface. Experimental rats were trained with four trials daily for 5 days during the acquisition phase. Rats facing a wall were put randomly placed into one of four different quadrants in the water maze and were allowed to swim to search for the underwater platform during acquisition training. Rats were permitted to stay for 10 s on the platform to survey the spatial cues in the environment before being returned to their cages. The path length, latency to reach the underwater platform, and mean swimming speed was recorded using a computerized video-tracking system. The underwater platform was removed after 5 days of acquisition and a spatial probe trial was performed at the sixth day. The time spent in the objective quadrant that previously contained the underwater platform during the spatial probe trial was calculated for 120 s.

### Nissl Staining

Rats were deeply anaesthetized with chloral hydrate (350 mg/kg, i.p.) and the thoracic cavity was cut rapidly to expose the heart. A catheter was inserted into the ascending aorta. Rats were perfused with 4% paraformaldehyde (PFA, pH7.4) in phosphate-buffered saline following systemic blood clearance by heparin saline. The brains were removed and fixed in 4% PFA overnight at 4°C, and were then dehydrated, transparentized, and embedded in paraffin. Serial coronal sections were gained by a rotary microtome (RM 2016; Shanghai Leica). All hippocampal sections were gathered in sequence. Nissl staining was employed to assess hippocampal structure. Briefly, hippocampal sections were deparaffinized, rehydrated, and stained with 1% toluidine blue. Every 15th stained section was chosen for quantitative analysis (n = 3 rats per group, five sections per rat). Images of CA1 and CA3 in hippocampal sections were acquired with 90× magnification. Neurons were measured and quantified using ImageJ software.

### Immunohistochemistry

Paraffin sections were used for immunohistochemistry. Serial coronal sections of brain were gained and hippocampal sections were gathered in sequence. Every 15th staining section was chosen for quantitative analysis (five sections per rat). After being dewaxed in xylene, hippocampal sections were hydrated in a graded series of alcohols. Then the sections placed into 0.01 M citrate buffer (pH6.0) were heated for 8 min in microwave oven to complete antigen retrieval, subsequently were blocked for endogenous peroxidases. The treated hippocampal sections were sealed using 3% BSA for 30 min at room temperature, and were then incubated with an anti-α7nAChR antibody (0.5 mg/ml, Abcam, Cat. ab110851, United States ) overnight at 4°C. After washing three times, the sections were incubated with a biotinylated anti-rabbit IgG (1:1,000, Abcam, Cat.150,077, United States ) for 1 h at 37°C and were then developed via DAB reagent. Subsequently, the sections were counterstained with hematoxylin, dehydrated, and mounted on gelatin-coated slides. Images of CA1 and CA3 regions were obtained with 90× magnification. Three fields in one section were chosen for calculating the numbers of positive neurons. Positively-labeled neurons were quantified using ImageJ software.

### Golgi Staining

The brains were removed and fixed in 4% PFA for 48 h, and were then cut into 2**-**3-mm-thick sections, after which they were immersed in Golgi dye solution for 14 days in a cool and ventilated condition. During this period, Golgi dye solution was changed every 3 days. The brain sections were dehydrated with a gradient sucrose solution in the dark at 4°C for 3 days. Subsequently, the brain sections were developed with strong ammonia water for 45 min and were immersed into acid-hardening fixative for 45 min. The brain sections were dehydrated once again with 30% sucrose in the dark at 4°C for 2–3 days. After embedding in OCT, the brain sections were cut into frozen sections (100 µm thick) and mounted with glycerin gelatin. Images of hippocampal sections were obtained via a digital slice scanner. There is evidence that terminal branches are more plastic than non-terminal branches, so numbers of terminal branches of dendrite were used to evaluated localized dendritic remodeling (Wellman, 2017). Dendritic segments (25 µm in length each) of 10 neurons per animal were selected in the region of the dentate gyrus for counting numbers of dendritic spines. Densities of dendritic spines were expressed as spines per unit length.

### Measurement of Neurotransmitters

Hippocampus tissues were homogenized with ice-cold phosphate-buffered saline and centrifuged at 12,000 rpm for 10 min at 4°C to obtain supernatants. Glutamate and ACh levels were measured using colorimetry according to the manufacturer’s instructions (Nanjing Jian Cheng Bioengineering Institute, China). For the determination of glutamate levels, supernatants were catalyzed by glutamate dehydrogenase, which produced α-ketoglutarate and reduced NADH, which have high absorption peaks. Sample absorbance was read at 340 nm. Measurement of ACh was accomplished via the hydroxylamine method. Briefly, supernatants were incubated with hydroxylamine and then reacted to ferric ion, which produced a red-purple complex. The absorbance was measured at 550 nm. The contents of GABA were assessed using commercial ELISA kits according to the manufacturer’s instructions (Cloud-Clone Corp, United States ). Briefly, the sample was incubated with Reagent A for 1 h at 37°C. After washing, Reagent B was added and incubated for 30 min at 37°C, after which the sample was developed with substrate solution. Absorbance was measured at 450 nm using a microplate reader.

### Western Blotting

Hippocampi were separated, homogenized with ice-cold RIPA buffer containing 1% PMSF, and centrifuged at 12,000 g for 10 min at 4°C. The supernatant was collected and the protein concentration was measured using a BCA kit (Beyotime, Shanghai, China). Protein samples (30 μg each) were isolated by 10% SDS-PAGE and transferred to PVDF membranes (Millipore, United States ). After blocking with 5% nonfat milk in Tris-buffered saline containing Tween 20 (TBST) at room temperature for 1 h, membranes were incubated with anti-α7nAChR (1:500, Abcam, Cat.10096, United States), anti-GLT-1 (1:1,000, Invitrogen, Cat.701,988, United States), and anti-SYP (1:1,000, Servicebio, Cat.11553, China) antibodies overnight at 4°C. The membranes were washed three times with TBST and were then subsequently incubated with HRP-conjugated secondary antibodies in TBST at room temperature for 1 h. The membrane was dyed with ECL reagent and developed using autoradiography film. Protein-band intensities were quantified using Quantity One software (Bio-Rad).

### Statistical Analyses

The experimental data were analyzed by GraphPad Prism and are expressed as the mean ± standard error of the mean (SEM). Homogeneity of variances among groups was evaluated using one-way analysis of variance (ANOVA). The Bonferroni method was applied for multiple comparisons of means between two groups. Differences were considered statistically significant at *p* < 0.05.

## Data Availability

The original contributions presented in the study are included in the article/Supplementary Material, further inquiries can be directed to the corresponding authors.

## Abbreviations

TLE, temporal lobe epilepsy; SRS, spontaneous recurrent seizures; SE, status epilepticus; AEDs, anti-epileptic drugs; CNS, central nervous system; ACh, acetylcholine; α7nAChRs, α7 nicotinic acetylcholine receptors; GABA, γ-aminobutyric acid; α-bgt, alpha-bungarotoxin; JME, juvenile myoclonic epilepsy; ADNFLE, autosomal-dominant nocturnal frontal lobe epilepsy; PFA, paraformaldehyde; GLT-1, glutamate transporter-1.
