# Supplementary figures and images for: Cytisine Exerts an Anti-Epileptic Effect via α7nAChRs in a Rat Model of Temporal Lobe Epilepsy
Source: Front Pharmacol. 2021 Jun 24;12:706225. doi: 10.3389/fphar.2021.706225 (PMC8263902; doi:10.3389/fphar.2021.706225)

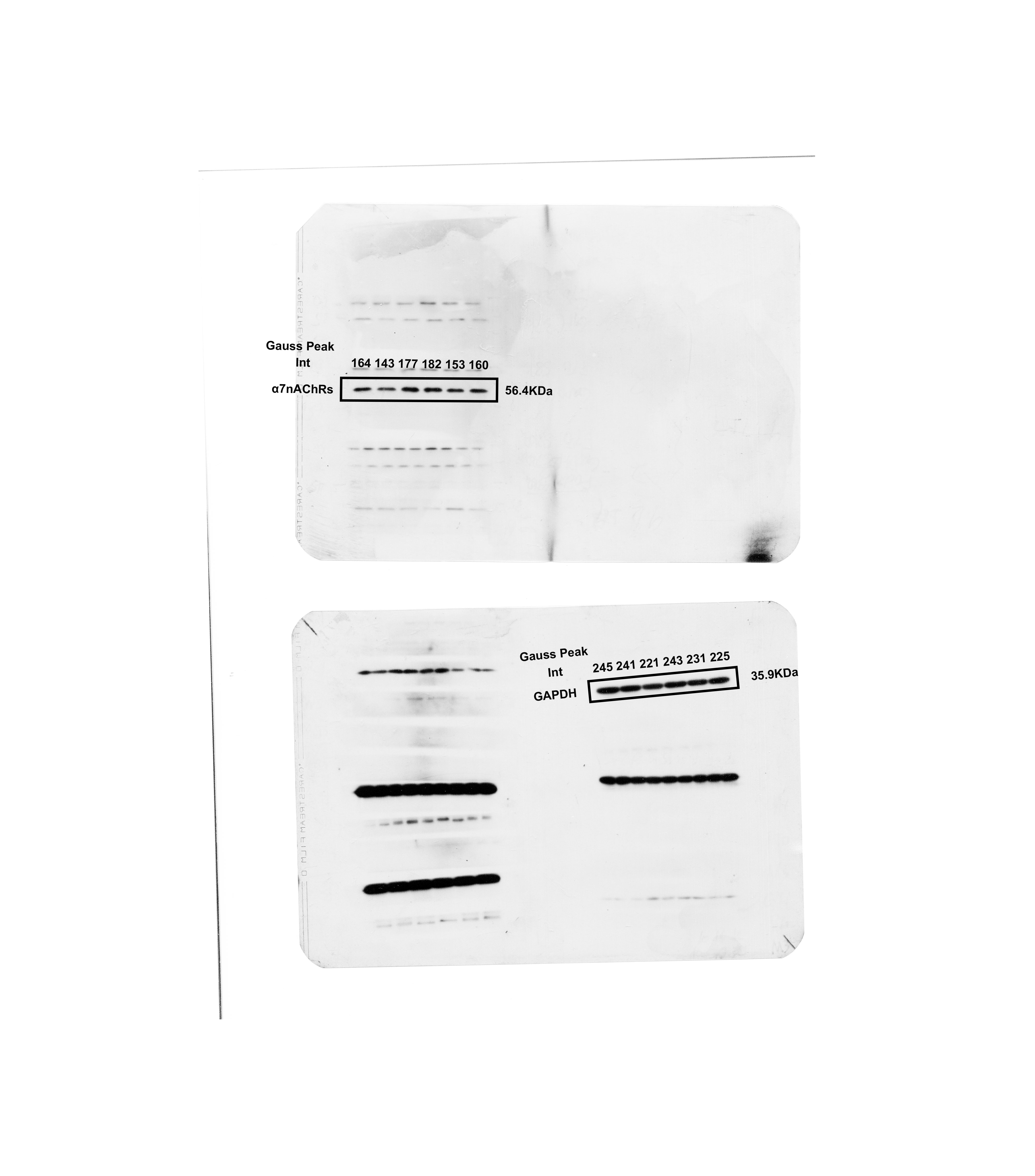

Supplement: Supplementary file 1 [file Image3.JPEG]

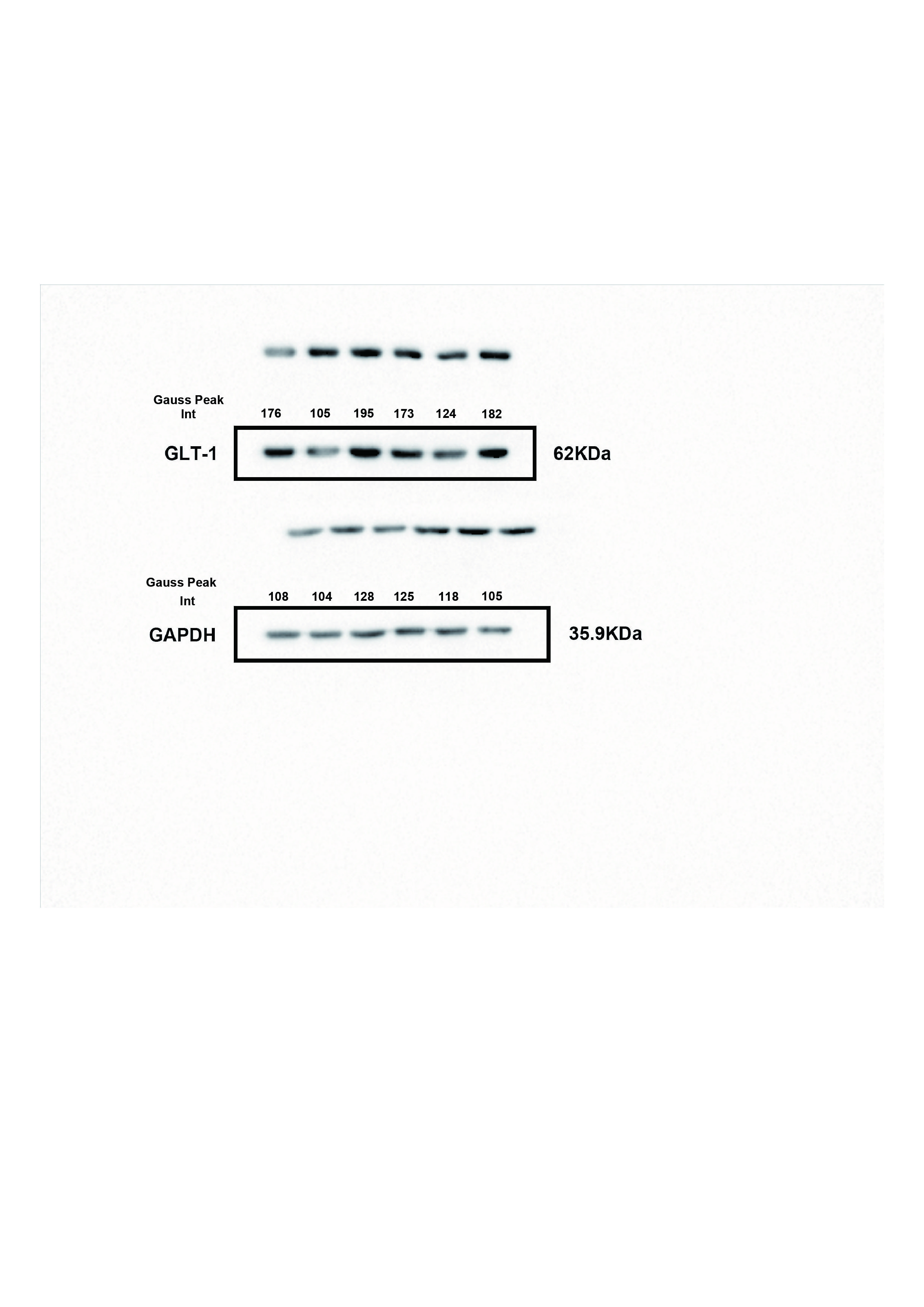

Supplement: Supplementary file 2 [file Image1.JPEG]
